# HPV Prevalence and Predictive Biomarkers for Oropharyngeal Squamous Cell Carcinoma in Mexican Patients

**DOI:** 10.3390/pathogens11121527

**Published:** 2022-12-13

**Authors:** Diego Octavio Reyes-Hernández, Adriana Morán-Torres, Roberto Jimenez-Lima, Ana María Cano-Valdez, Carlo César Cortés-González, Leonardo Josué Castro-Muñoz, Leslie Olmedo-Nieva, Silvia Maldonado-Frías, Nidia Gary Pazos-Salazar, José de Jesús Marín-Aquíno, Alejandro García-Carrancá, Adela Carrillo-García, J. Omar Muñoz-Bello, Marcela Lizano, Joaquín Manzo-Merino

**Affiliations:** 1 Unidad de Investigación Biomédica en Cáncer, Instituto Nacional de Cancerología, Mexico City 14080, Mexico; 2 The Wistar Institute, Philadelphia, PA 19104, USA; 3 División de Estudios de Posgrado e Investigación, Facultad de Odontología, Universidad Nacional Autónoma de México, Mexico City 04360, Mexico; 4 Facultad de Ciencias Químicas, Benemérita Universidad Autónoma de Puebla, Puebla City 72570, Mexico; 5 Departamento de Patología, Hospital Dr. Manuel Gea González, Mexico City 14080, Mexico; 6 Departamento de Medicina Genómica y Toxicología Ambiental, Instituto de Investigaciones Biomédicas, Universidad Nacional Autónoma de México, Mexico City 04510, Mexico; 7 Cátedras CONACyT-Instituto Nacional de Cancerología, Mexico City 14080, Mexico

**Keywords:** HPV, OPSCC, p16, biomarker, Mexican population

## Abstract

Background: Worldwide prevalence of Oropharyngeal Squamous Cell Carcinoma (OPSCC) has increased, affecting mostly young males. OPSCC associated with Human Papillomavirus (HPV) infection exhibits particular characteristics in terms of response to treatment, hence HPV has been proposed as a prognostic factor. The impact of HPV positivity and associated biomarkers on OPSCC in the Mexican population has not been addressed. Therefore, the analysis of OPSCC prognostic markers in the Mexican population is necessary. Methods: Retrolective study in Mexican OPSCC patients, where HPV prevalence, p16 and EGFR levels were assessed using INNO-LiPA and immunohistochemistry. Results: We found an HPV prevalence of 57.6% in OPSCC cases treated at a reference center in Mexico. HPV and p16 positivity, as well as EGFR, associate with better outcomes in OPSCC patients, and they also promote reduced death risk. Notably, HPV presence and p16 positivity showed a significant association with disease-free survival (DFS), with a HR of 0.15 (*p* = 0.006) and a HR of 0.17 (*p* = 0.012), respectively, indicating a possible role as predictive biomarkers in Mexican OPSCC patients. Conclusions: Our results reflect the clinical utility of p16 analysis to improve overall survival (OS) and to predict recurrence in oropharyngeal cancer. These results position p16 and HPV as predictive biomarkers for OPSCC.

## 1. Introduction

Head and neck cancer (HNC) is a public health threat, representing the seventh most common neoplasia with approximately 562,328 annual cases [1]. HNC has been associated with alcohol and tobacco consumption traditionally. Nonetheless, Human Papillomavirus (HPV) infection has been recognized as an important etiological factor for HNC development in the past years, mainly affecting the oropharyngeal region [2]. Particularly, oropharyngeal squamous cell carcinoma (OPSCC) has gained attention in past decades due to the alarming increase in numbers, especially in HPV-associated cases [3,4]. HPV-positive HNC is associated with the practice of oral sex, mainly affecting young men. The presence of HPV confers better prognosis after a radiotherapy treatment and a better overall survival (OS) [5].

An improvement in OS has been described for HPV-positive cancers, as determined by detecting HPV DNA or by analysis of subrogate marker, p16, expression by immunohistochemistry (IHC) [6]. Recently, the RTOG-0234 group presented the results of a retrospective analysis of clinical trials 0129 and 0522, where the utility of p16 as a predictive factor was found, indicating that positivity to this biomarker not only predicts OS, but also recurrence-free survival (RFS), thus promoting the value of p16 identification in patients with HNC [7]. Although the evidence indicates that p16 could be a good prognostic factor in HNC, its potential utility, together with the presence of HPV, has yielded controversial results, indicating that these markers should be further explored in different populations [8,9,10].

Moreover, several proteins are associated with improved survival in HNC. For instance, the epidermal growth factor receptor (EGFR) is found to be overexpressed in head and neck squamous cell carcinomas (HNSCC) [11], mostly in HPV-negative cases [12,13,14]. In the majority of these tumors, this receptor is overexpressed or harbors mutations [15], representing a drug target that provides therapeutic opportunities for these patients [16]. However, the search for additional biomarkers for HNC is still underway.

The prevalence of HPV in HNC in Mexico and biomarker application studies are currently limited. In a retrospective case series of HNC of Mexican patients, the prevalence of HPV ranged from 22.3 to 42%. Specifically, for the oral cavity, 17.2–18.75% has been reported, while in oropharyngeal cancer, the prevalence ranges from 40.5–68.75%, and a low proportion has been identified for the larynx, ranging between 6.25 to 18.2% [17,18,19]. However, the potential clinical application of the presence of HPV or the expression of additional subrogate biomarkers, particularly for OPSCC, has been poorly explored in the Mexican population, considering the possible increase in this neoplasm in the coming years. Thus, we aimed to describe the prevalence of HPV, as well as the clinical features of OPSCC patients in a national reference institution in Mexico, and to determine the potential utility of HPV presence and p16 positivity as prognostic biomarkers in the Mexican population.

## 2. Materials and Methods

### 2.1. Clinical Specimens and Patient Characteristics

This retrospective study analyzed biopsies from OPSCC patients treated at the National Cancer Institute of Mexico. We obtained data from patients who received treatment between 2000 to 2017, and their respective clinical and sociodemographic characteristics were collected. The study protocol was approved by the Ethics (CEI/998/15) and Scientific (015/039/IBI) Institutional review boards and followed the guidelines of the Declaration of Helsinki.

The present cohort study comprised 66 cases, selected for meeting the following criteria: existence of formalin-fixed paraffin-embedded (FFPE) primary tumor material prior to treatment with curative intent, histologically proven squamous cell carcinoma (SCC) originated from the tonsillar portion, and different stages were included according to the 7th TNM edition for oropharyngeal cancer (I, II, III, IV and IVa). Medical records were used to collect demographic and clinical features. Cases treated for metastatic and palliative treatment were excluded, as well as those lacking complete data.

### 2.2. Histological Evaluation

FFPE tumor blocks were obtained from the institutional pathology bank. Subsequently, 2 μm-thick tissue sections were obtained. To verify the diagnosis of SCC and demarcate the malignant cells, evaluation of H&E tissue slides was performed by a pathologist specializing in head and neck cancer

### 2.3. Immunohistochemistry

Tissue sections from 66 patients were used for the IHC staining, using the p16 mouse monoclonal antibody (ROCHE^®^) (Roche Holding AG, Basel, Swiss). The Ventana Benchmark LT automated immunostainer (ROCHE^®^) was used following the standard protocol for p16 [20]. Appropriate tissue staining controls were used routinely. A p16 IHC was considered positive if the tumor section had strong and diffuse nuclear and cytoplasmatic staining in >70% of malignant cells [21]. EGFR IHC was performed with anti-EGFR antibody (Abcam^®^) (Abcam Biotechnology company, Cambridge, UK) using the Mouse/Rabbit Polydetector DAB HRP Brown System (Bio SB^®^) ( Bio SB, Inc., Goleta, CA, UEA), according to the manufacturer’s instructions. The level of expression was assessed by an expert pathologist, and data were categorized into high or low expression.

### 2.4. DNA Extraction and HPV Genotyping

Ten μm-thick tissue sections were obtained from the FFPE blocks and then employed for DNA purification using the DNeasy Blood & Tissue Kit (QIAGEN^®^) (QIAGEN, Hilden, Germany) according to manufacturer’s instructions. DNA samples were quantified in a full spectrum Nanodrop™ spectrophotometer (ThermoScientific^®^) (Thermo Fisher Scientific, Waltham, MA, UEA) and further utilized for HPV identification using the INNO-LiPA^®^ HPV Genotyping Extra II (Fujirebio^®^) (Fujirebio, Tokyo, Japan) assay. This test is based on reverse hybridization after highly sensitive PCR amplification with SPF10 primers that allow the detection of 32 HPV genotypes simultaneously [22]. For each test, internal routine controls were used.

### 2.5. Statistical Analysis

Data were collected and descriptive statistics were used to summarize the demographic and clinical features. The OS and disease-free survival (DFS) estimations were made using the Kaplan–Meier method according to positiveness or protein levels. Additionally, the Log-Rank test was employed to make comparisons between tests. Hazard Ratios (HR) were calculated for each group if statistical significance was achieved. *p* ≤ 0.05 was considered as statistically significant. All statistical analyses were carried out using SPSS^®^ V.21 (IBM Corp., Armonk, NY, USA).

## 3. Results

### 3.1. Population Description and HPV Prevalence

A total of 66 cases of OPSCC that met the inclusion criteria were included. The demographic and pathological characteristics are described in Table 1. Most of the study population were men (78.7%) with a median age of 60.67 years; 43.9% had only elementary school education. Alcohol and tobacco consumption were present in 66.7% and 71.2% of the patients, respectively. Advanced stages (IVA, IVB) represented 39.4% of the cohort.

Chemoradiation was the most common therapy regimen (40.9% of cases), and 23 cases (34.8%) presented complete clinical response, among which 11 cases presented disease recurrence.

57.6% of the cases were positive for HPV, with HPV16 the most prevalent genotype in 28 cases (73.68%). In addition, the presence of other HPV types, including HPV 6, 11, 18, 53 and 59, were detected as coinfections with HPV16, and 3 cases were positive for both HPV11 and 16. The median age of HPV-positive cases was 60.64 years, while HPV-negative cases exhibited a median age of 60.67 years.

### 3.2. Protein Expression and HPV Association

The expression patterns of p16 and EGFR proteins were evaluated by immunohistochemistry. Each antibody was validated on positive tissues prior to evaluating OPSCC cases. Protein levels were classified as positive or negative for p16 and high or low for EGFR, based on expression. A total of 27 cases were classified as p16 positive (40.9%), while 12 cases were EGFR high (41.4%).

In addition, association tests were performed to determine whether viral presence influenced the expression of the analyzed proteins. We determined that there was no statistically significant association between the presence of HPV and EGFR (data not shown). However, the presence of HPV is significantly associated with p16 expression (*p* = 0.008), as previously shown [23].

### 3.3. Association of Viral Presence, EGFR and p16 Levels with OS in Mexican Patients

**Figure 1:**
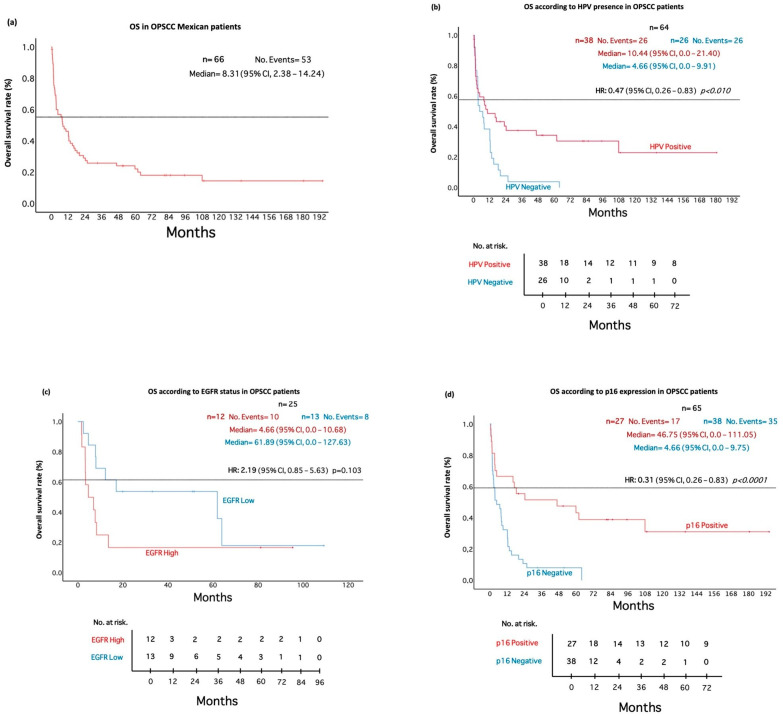
Overall survival in OPSCC patients according to HPV, p16 and EGFR. (**a**) Overall survival of the analyzed cohort. (**b**) HPV presence improves OS in OPSCC Mexican patients. (**c**) p16 impacts OS in OPSCC patients. (**d**) High EGFR levels are associated with poor OS in OPSCC Mexican patients.

We proceeded to analyze the impact of EGFR and p16 protein levels, as well as viral presence, on OS. First, the OS time in the analyzed cohort was calculated, resulting in a median of 8.31 months (95% CI: 2.38–14.24) (Figure 1A). Subsequently, the impact of the presence of HPV on OS was determined, resulting in viral presence significantly associated with an increase in OS *(p* = 0.008), where positive cases exhibit a median survival of 10.44 months (95% CI: 0.0–21.40), compared to HPV-negative cases with only 4.66 months (95% CI: 0.0–9.91) (Figure 1B). Furthermore, when performing Cox regression analysis, viral presence provides a 53% risk reduction for death with a HR (Hazard ratio) of 0.47 (95% CI: 0.26–0.83) (*p* = 0.010).

When analyzing OS with respect to p16 and EGFR levels, both proteins were found to have an impact on patient OS. High EGFR expression had a significant impact on OS *(p* = 0.024), predicting an unfavorable outcome, with the high expression group showing a median of only 4.66 months (95% CI: 0.0–10.68), while patients with low EGFR expression had a median of 61.89 months (95% CI: 0.0–127.63) (Figure 1C). The estimated median survival for p16-negative patients was 4.66 months (95% CI: 0.0–9.75), while for those who were positive for p16, it was 46.75 months (95% CI: 0.0–111.05). A statistically significant difference was found (*p* ≤ 0.0001) when performing the Cox proportional hazards analysis for p16 levels, with a HR of 0.31 (95% CI: 0.26–0.83). In other words, there is a 69% reduction in the risk of death with positive p16 staining (Figure 1D).

### 3.4. Viral Presence and p16 Positivity Are Associated with DFS in OPSCC Mexican Patients

**Figure 2:**
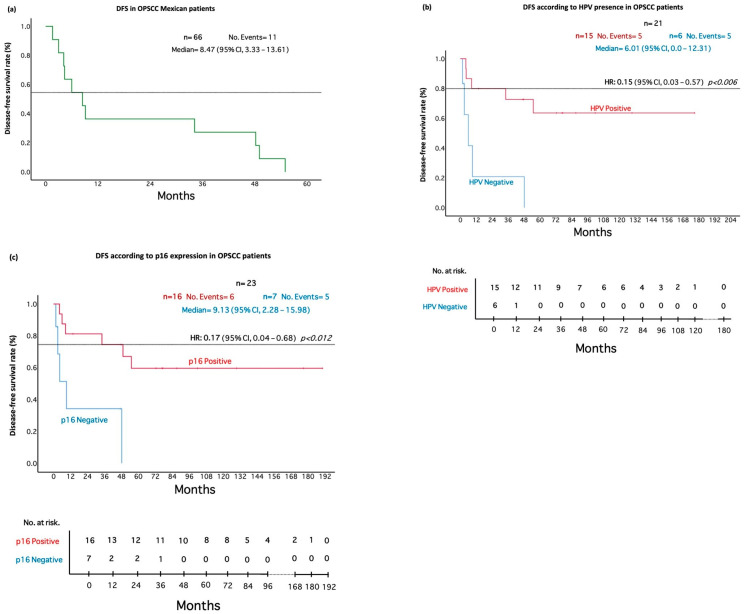
Disease-free survival in OPSCC patients according to HPV and p16 positivity. (**a**) Disease-free survival of the analyzed cohort. (**b**) HPV presence is associated with better DFS in OPSCC Mexican patients. (**c**) p16 predicts improved DFS in OPSCC Mexican patients.

A total of 23 cases had a complete response to treatment. Nevertheless, 11 of these cases presented recurrence of the disease (47.82%) during the first 9 months after clinical response. A median of 8.47 months for DFS was calculated for the population examined (Figure 2A). To assess the usefulness of p16 and the presence of HPV as predictive biomarkers, their association with DFS was tested.

The association between viral presence and DFS was analyzed, showing a median of 6.01 months for HPV-negative cases (95% CI: 0.0–12.31). While positive HPV cases did not reach the median, a fraction of 40% was free of disease after 60 months. A statistically significant difference (* *p* = 0.002) was found for viral presence and DFS (Figure 2B). This result strongly indicates that HPV positivity establishes a late appearance of recurrence. Cox proportional hazards analysis indicated a statistically significant difference (*p* = 0.006), obtaining a HR of 0.15 (95% CI: 0.03–0.57), which indicates that the presence of HPV reduces the risk of recurrence by 85%. Furthermore, when analyzing the performance of p16 for DFS, p16-negative cases exhibited a median DFS of 9.13 months (95% CI: 2.28–15.98), while positive cases did not reach a median; however, 60% of the patients were disease-free after 60 months. Importantly, we found p16 levels were statistically associated with DFS (*p* = 0.005), indicating that p16 positivity predicts late-onset recurrence (Figure 2C). Cox proportional hazards analysis indicated a statistically significant difference (*p* = 0.012) with a HR of 0.17 (95% CI: 0.04–0.68), showing that p16 positivity reduces the risk of recurrence by 83%.

## 4. Discussion

In recent years, OPSCC has shown an increase in incidence, especially in young men [5,24,25]. According to various reports, OPSCC has shown a greater increase than cervical cancer in the United States [3,4,26,27]. Although, this scenario has not yet been documented in Mexico, this possibility cannot be ruled out in the future because GLOBOCAN predictions estimate an increase of 100% in the coming years [28]. Thus, we aimed to analyze OPSCC cases in a Mexican population, determining HPV and potential biomarkers to support clinical management of patients and to improve therapeutic options with the ultimate goal of increasing therapeutic success.

We found a high prevalence of HPV in OPSCC (57.6%); in particular, HPV16 was detected in 73.68% of the positive cases. It has been stated that HPV-positive cases are associated with better prognosis [6], but the role of HPV in this phenomenon remains unclear. Although HPV positivity improves OS, its role in recurrence has only been explored in a couple of studies. In addition, there are cases of OPSCC that do not present an optimal response to treatment; therefore, we took on the task of exploring potential predictive biomarkers that could be useful in clinical practice.

In the present cohort, we found that men are the most affected by OPSCC (78.7%), which is consistent with other reports that associate this phenomenon with alcohol and tobacco consumption [5,29,30], a fact that was repeated in our cohort. Conversely, when analyzing the age at diagnosis, it was found that our patients had a median age of 60.91 years, including both HPV positive and HPV negative. Age over 60 is associated with HPV-negative cases, while HPV-positive cases exhibit a younger age at presentation [5,31]. Our results support the hypothesis that our population does not yet present the epidemiological transition towards young individuals. However, according to epidemiological predictions, an increase in the number of OPSCC cases is expected for our country, which could mean an increase in HPV-positive cases in the young population. In this regard, a recent work by our research group showed that young individuals exhibit a high prevalence of HPV infection (about 50%) in the oral cavity and oropharynx, presenting constant reinfections [32]. This fact may represent an important stimulus in cancer induction for future OPSCC cases.

One of the markers associated with the presence of HPV is the p16 protein, which acts as a surrogate marker for the effects of the E7 viral oncoprotein on pRb [33]. In the present work, p16 was found to be a potential prognostic and predictive biomarker, with a protective factor similar to that reported by Sturgis EM et al. and Harari et al. [34,35], although with lower median OS, probably because most of our cases were diagnosed in advanced clinical stages (IVA and IVB).

One of the factors associated with lower OS is the presence of disease recurrence after a successful treatment [36,37]. In our cohort, half of the patients with complete response were found to develop recurrence at a median of 54.99 months. Notably, only two reports have explored prognostic markers that help to predict recurrence events, thus our study lays the groundwork for using p16 as a marker capable of predicting the delayed onset of OPSCC recurrence in our population. To our knowledge, only Harari et al. (2014) and recently Bigelow et al. (2022) have reported similar results in randomized clinical trials (RTOG-0234) [7,35]. Particularly, these studies used combined therapy based on chemo-radiotherapy (Cisplatin or Docetaxel) plus Cetuximab, and their results indicate that p16 positivity confers a longer DFS time [7,35]. In this study, we propose that p16 could function as a recurrence marker, regardless of the treatment regimen. Although only one of the patients in our cohort received anti-EGFR therapy, the finding that high EGFR levels are associated with lower OS highlights the need to classify patients based on EGFR status, as they might be candidates for anti-EGFR therapies, which represents an opportunity to increase OS and DFS in patients with OPSCC in Mexico.

Our study presents some limitations regarding the number of cases included and the late stages at diagnosis. Nonetheless, we present solid data regarding HPV prevalence in the OPSCC Mexican population that could help to identify patients with better prognoses. Future studies are required to postulate additional biomarkers to improve OS, DFS and quality of life in Mexican patients.

## 5. Conclusions

We identified p16 expression as a biomarker for recurrence and OS in OPSCC Mexican patients. These results pose p16 as a predictive biomarker when HPV detection is not possible. Moreover, it was verified that HPV positivity has an impact on the OS of patients with OPSCC, whose pattern of protein expression could help in the improvement of therapies.

## Figures and Tables

**Table 1:** Population description.

Variable.	n = 66 (100%)
*Age median (±SD)*	
Both sexes	60.67 * (±11.78)
Men	59.48 * (±11.20)
Women	65.07 ** (±13.25)
*Sex*	
Men	52 (78.7%)
*Education* ^&^	
Illiterate	17 (25.8%)
Elementary school	29 (43.9%)
High school	11 (16.7%)
Superior school	7 (10.6%)
Undetermined	2 (3%)
*Comorbidities*	
No	48 (72.7%)
Yes	14 (21.2%)
Undetermined	4 (6.1%)
*History of alcoholism*	
Yes	44 (66.7%)
No	21 (31.8%)
Undeterminate	1 (1.5%)
*History of smoking*	
Yes	47 (71.2%)
No	18 (27.3%)
Undetermined	1 (1.5%)
*Clinical stage*	
II	3 (4.5%)
III	7 (10.6%)
IV A	17 (25.8%)
IV B	9 (13.6%)
Indeterminate	30 (45.5%)
*Therapeutic regimen*	
Surgery	10 (15.2%)
Chemotherapy	4 (6.1%)
Chemo-radiotherapy	27 (40.9%)
Radiotherapy	6 (9.1%)
Undetermined	19 (28.8%)
*Clinical response*	
Complete	23 (34.8%)
Partial	8 (12.1%)
Stable disease	5 (7.6%)
Progression	3 (4.5%)
Undetermined	5 (7.6%)
No data	22 (33.3%)
*Recurrence*	n = 23 (100%)
(Only complete response)	
No	12 (52.18%)
Yes	11 (47.82%)
*HPV*	n = 66 (100%)
Positive	38 (57.6%)
Negative	26 (39.4%)
Undetermined	2 (3.0%)
*p16*	n = 66 (100%)
Positive	27 (40.9%)
Negative	38 (57.6%)
Indeterminate	1 (1.5%)
*EGFR (Intensity)*	n = 29 (100%)
High	12 (41.4%)
Low	13 (44.8%)
Undetermined	4 (13.8%)

* Kolmogorov–Smirnov normality test; Global: *p* = 0.041; Men: *p* = 0.015; ** Shapiro–Wilk normality test: Women: *p* = 0.816; ^&^ Elementary: 6–12 yo; High school: 12–18 yo; Superior school: 18 yo and on.

## Data Availability

The data that support the findings of this study are available from the corresponding author upon reasonable request.
